# Regulation of p53: a collaboration between Mdm2 and MdmX

**DOI:** 10.18632/oncotarget.443

**Published:** 2012-03-10

**Authors:** Dongsheng Pei, Yanping Zhang, Junnian Zheng

**Affiliations:** ^1^ Laboratory of Biological Cancer Therapy, Xuzhou Medical College, Xuzhou, China; ^2^ Department of Radiation Oncology and Lineberger Comprehensive Cancer Center, School of Medicine, the University of North Carolina at Chapel Hill, Chapel Hill, NC, USA

**Keywords:** Mdm2, MdmX, p53, cancer

## Abstract

p53 plays an important role in the regulation of the cell cycle, DNA repair, and apoptosis and is an attractive cancer therapeutic target. Mdm2 and Mdmx are recognized as the main p53 negative regulators. Although it is still unknown why Mdm2 and Mdmx both are required for p53 degradation, a model has been proposed whereby these two proteins function independent of one another; Mdm2 acts as an E3 ubiquitin ligase that catalyzes the ubiquitination of p53 for degradation, whereas Mdmx inhibits p53 by binding to and masking the transcriptional activation domain of p53, without causing its degradation. However, Mdm2 and Mdmx have been shown to function collaboratively. In fact, recent studies have pointed to a more important role for an Mdm2/Mdmx co-regulatory mechanism for p53 regulation than previously thought. In this review, we summarize current progress in the field about the functional and physical interactions between Mdm2 and Mdmx, their individual and collaborative roles in controlling p53, and inhibitors that target Mdm2 and Mdmx as a novel class of anticancer therapeutics.

## INTRODUCTION

The p53 tumor suppressor plays a pivotal role in regulating cellular processes including cell cycle arrest, apoptosis, cell metabolism and senescence. Mutation of the *TP53* gene or inactivation of the p53 signaling pathway occurs at a high frequency in many human tumors, suggesting that p53 plays a critical role in preventing normal cells from becoming cancerous. p53 is a stress-inducible protein; it is inactive under normal physiological conditions and activated in response to various types of stresses such as DNA damage and ribosomal stress [1]. Activated p53 can either induce cell cycle arrest and inhibit cell growth or promotes cell apoptosis depending on different type of stress and the cellular context. Multiple mechanisms have been revealed to collectively accomplish the regulation of p53 activity [2,3], which ultimately determines the selectivity of p53 for specific transcriptional targets, resulting in precise control of the p53 activity.

p53 is the most frequently inactivated tumor suppressor gene in human cancer. Clinical studies have shown that p53 is mutated in approximately 50% of human cancers. Mdm2 and MdmX (also known as MDM4) are two structurally related proteins that play a critical role in downregulating p53 activity in embryonic cells and stem cells under normal conditions [4]. Therefore, the amplification and/or aberrant expression of Mdm2 and MdmX occur in a number of tumors of diverse origin, especially in tumors that retain wild-type p53.

Mdm2 (*m*urine *d*ouble *m*inute *2*) was discovered on double minute chromosomes in a derivative cell line of NIH-3T3 cells [5,6]. Mdm2 belongs to the family of E3 ubiquitin ligases that contain a RING [*r*eally *i*nteresting *n*ew *g*ene] domain [7] and serves as the major E3 ubiquitin ligase for p53 degradation. Several studies have illustrated the importance of Mdm2 in the control of p53 activity. The mechanism by which Mdm2 suppresses p53 has classically been thought to occur by two distinct ways: by binding to the N-terminal domain of p53 and masking p53's access to transcriptional machinery, and by ubiquitinating p53 and targeting it for proteasomal degradation [8-11]. However, recent research has shown that Mdm2-p53 binding alone in the absence of Mdm2 E3 ubiquitin ligase activity is insufficient to suppress p53 activity [12].

MdmX has been identified as a highly homologous gene that is closely related to Mdm2 [13,14]. Similarly to Mdm2, MdmX possesses a p53 binding domain at its N-terminus and a RING finger domain at its C-terminus through which it heterodimerizes with Mdm2. However, unlike Mdm2, MdmX does not have appreciable ubiquitin ligase activity. Because of its sequence similarity with Mdm2 and its ability to inhibit p53-induced transcription when overexpressed, MdmX has been hypothesized to act as a negative regulator of p53 through physical binding [15].

## MODELS FOR THE REGULATION OF P53 BY MDM2 AND MDMX

Genetic evidences have shown that Mdm2 and MdmX are the two essential negative regulators of p53, since the concomitant deletion of p53 can rescue the embryonic lethality caused by the deletion of either Mdm2 or MdmX. The fact, neither Mdm2 nor MdmX can compensate for one another *in vivo* to inhibit p53 suggests that Mdm2 and MdmX perform critical, non-overlapping functions in p53 suppression. The requirement of both Mdm2 and MdmX raises the question as to why p53 needs two highly similar regulators. A proposal has been put forward whereby these two homologous proteins can function independently: Mdm2 functions as an E3 ubiquitin ligase that catalyzes the ubiquitination of p53, MdmX, and itself for proteosomal degradation [16-18], whereas MdmX functions mainly by binding to and masking the transcriptional activation domain of p53.

Mdm2 and MdmX physically interact with and functionally affect each other. Mdm2 can form a homodimer *in vitro*, but it is also capable of forming a more stable heterodimer with MdmX through their RING domains [19,20]. *In vitro* transfection studies have indicated that MdmX stabilizes Mdm2 by interfering with Mdm2 autoubiquitination. However, MdmX has also been reported to be ubiquitinated and degraded by Mdm2 [18]. Other studies have shown that MdmX is able to inhibit Mdm2-mediated p53 degradation by competing with Mdm2 for p53 binding resulting in the accumulation of p53 [20-22].

Many lines of evidence point to an intricate interaction that exists between Mdm2 and MdmX in p53 regulation [23-27]. Mdm2 and MdmX proteins are found to exist in cells predominantly in the form of a heteroduplex [25], and structural studies have predicted that the formation of an Mdm2-MdmX heteroduplex is structurally favored over the formation of homoduplexes of either protein [28]. It has been shown that Mdm2 alone is a relatively inefficient E3 ubiquitin ligase [25], but becomes more efficient at ubiquitinating p53 after heterodimerization with MdmX [26]. A previous genetic study in the development of the mouse central nervous system (CNS) has revealed a synergistic role between Mdm2 and MdmX, as well as independent functions of Mdm2 and MdmX for p53 inhibition. In this study, mice lacking Mdm2 in the CNS developed hydranencephaly at embryonic day 12, whereas mice in which MdmX was deleted in the CNS showed a proencephaly phenotype at embryonic day 17.5. Interestingly, the simultaneous deletion of both genes resulted in an even earlier and more severe CNS phenotype. All of these phenotypes were rescued by the concomitant deletion of p53. These observations strongly support a synergistic relationship between Mdm2 and MdmX in the inhibition of p53 activity during the development of the CNS [29]. Based on both *in vitro* and *in vivo* studies, another, perhaps more convincing model was proposed in which Mdm2 and MdmX work together to control p53 activity [30,31].

In order to determine whether Mdm2-p53 binding alone is sufficient to suppress p53 activity, or whether Mdm2-mediated ubiquitination is also required in that regard, Itahana *et al.* [12] generated knock-in mice harboring a single point mutation (C462A) in one of the zinc-coordinating residues in the C-terminal RING domain of Mdm2 that is critical for the E3 ubiquitin ligase activity of Mdm2. The homozygous C462A mutation was embryonic lethal and the lethality was rescued by the concomitant deletion of p53, providing evidence that the Mdm2 RING domain is required for the regulation of p53 activity *in vivo*. This study used an inducible p53^ER^ system that allowed the investigators to induce the expression of p53 *ex vivo* in mouse embryonic fibroblast (MEF) cells to study the interactions of p53 in the Mdm2 mutant background. Upon induction of p53 in MEF cells, Itahana *et al.* demonstrated that the Mdm2 RING mutant protein, although deficient in the ability to ubiquitinate p53, is fully capable of binding to p53 proving that Mdm2 cannot suppress p53 transcriptional activity through binding alone. However, the authors also showed that the C462A mutation alters the structure of the Mdm2 RING domain to the extent that the Mdm2 C462A mutant is unable to heterodimerize with MdmX. Therefore, the study cannot explain whether Mdm2 and MdmX interaction is required for p53 suppression.

Recently, two studies using MdmX RING domain mutant knock-in alleles demonstrated that the RING domain of MdmX, like that of Mdm2, is also critical for regulating p53 activity during early embryogenesis [32,33]. In the Huang *et al.* study, mice harboring an MdmX C462A mutation in one of the critical zinc-coordinating residues of the RING domain died at approximately day 9.5 of embryonic development as the result of an increase in apoptosis and a decrease in cell proliferation. The concomitant deletion of p53 completely rescued the embryonic lethality of the MdmX C462A mutation [32]. Importantly, the authors showed that the MdmX C462A mutant protein does not bind to Mdm2, yet it retains the ability to bind to p53 to the same degree as wild-type MdmX. These results indicate that even though both Mdm2 and MdmX are fully capable of binding to p53 individually, the disruption of the Mdm2-MdmX heterocomplex causes p53 activation *in vivo*. In a similar study performed by Pant *et al.*, the authors used a tamoxifen-based Cre-inducible MdmX ΔRING allele to investigate the role of Mdm2-MdmX heterodimerization in Mdm2 and p53 regulation. They found that although the heteroduplex is essential during embryonic development, heterodimerization is dispensable during the adult life of the mouse. Together, these studies provide compelling evidence that the action of the heterodimer of Mdm2 and MdmX, and not necessarily the independent action of either protein is crucial to the appropriate control of p53. However, these studies cannot answer a remaining question as whether the Mdm2 E3 ubiquitin ligase function is still required for p53 suppression, because the Mdm2 RING mutation simultaneously disrupts its E3 ligase function and its binding to MdmX, and because *in vitro* studies have shown that Mdm2 by itself is a relatively weak E3 for p53 degradation and its heterodimerization with MdmX enhances its E3 activity [25,26]. Thus, the generation and analysis of mutations that block the ubiquitin ligase activity but do not affect the heterodimerization between Mdm2 and MdmX *in vivo*, if technically possible, would be essential for understanding the importance of the *in vivo* cooperation between Mdm2 and MdmX.

## THE MDM2/MDMX RATIO DETERMINES P53 STABILITY AND ACTIVITY

Although Mdm2 and MdmX have a synergistic relationship that effectively inhibits p53, as discussed above, Mdm2 and MdmX also have independent roles in the regulation of p53. MdmX can inhibit p53 transcriptional activity by interfering with the ability of p53 to interact with the basal transcription machinery, while Mdm2 can target p53 for degradation. Several studies have reported that elevated MdmX levels stabilize p53 by inhibiting Mdm2-mediated p53 degradation without interfering significantly with Mdm2-dependent p53 ubiquitination [21,22,34,35]. Transfection studies have also provided evidence that MdmX can stabilize Mdm2 by interfering with auto-ubiquitination and degradation of Mdm2 [34]. Conversely, results from Linares *et al.* have shown that MdmX stimulates Mdm2-mediated ubiquitination of p53, as well as Mdm2 self-ubiquitination *in vitro* [26]. These inconsistent data reflect the complex relationship between Mdm2 and MdmX and are often difficult to reconcile because of the nature of *in vitro* overexpression studies. Quantitative analysis has demonstrated that the level of endogenous MdmX is present at different proportion to that of Mdm2 in several types of human cell lines [36]. This observation might well account for the discrepancies when trying to examine the effect by altering the MdmX abundance in various *in vitro* studies and also indicates the relative level of Mdm2 and MdmX is crucial for controlling p53 stability and activity, which was further demonstrated by the recent crystal structures studies. Linker *et al.* revealed that the primary and secondary interfaces in Mdm2 homodimers or Mdm2/MdmX heterodimers are crucial for the binding of ubiquitin E2 enzyme and ubiquitylation of the subunit. Because Mdm2 homodimers have two primary and secondary interfaces for ubiquitin E2 enzyme binding and the E2 enzyme can be recruited by either monomer, which will lead to the ubiquitylation of the other subunit. However, in the Mdm2/MdmX heterodimer, only Mdm2 can provide the primary E2 interaction site while the secondary interface will be provided by MdmX, which will not cause the ubiquitylation and degradation of Mdm2. Therefore, the ratio of Mdm2/MdmX can be used to explain Mdm2 status in different situations: Mdm2 will form homodimer and degrade by itself through ubiquitination if the ratio is high, on the contrary, Mdm2 will be stabilized if the ratio is low [37].

Both *in vivo* and *in vitro* experiments have demonstrated that p53 can bind to p53-responsive elements located within the Mdm2 gene and promote its transcription thereby set up a negative feedback regulatory loop [38,39], while in contrast p53 cannot transactivate MdmX. Because of this, the protein level of Mdm2 fluctuates widely upon p53 activation, whereas since MdmX is not a p53 transcriptional target, the level of MdmX remains relatively constant. Thus, the stress-induced up-regulation of p53 increases the levels of Mdm2 thereby modulating the ratio of Mdm2 to MdmX and serving as a negative feedback loop by which p53 can regulate itself. When the level of MdmX is higher than the level of Mdm2, MdmX will inhibit Mdm2-mediated p53 degradation resulting in the stabilization of the level of Mdm2 through the stabilization of p53. In this proposed feedback loop, MdmX acts as a sensor of the concentration of Mdm2 and controls the balance between Mdm2 and p53.

## MDM2 AND MDMX IN P53 UBIQUITINATION

It has been widely accepted that Mdm2 antagonizes p53 by promoting its ubiquitination and proteasome-dependent degradation [8,10]. In addition to the polyubiquitin-dependent degradation of p53, Mdm2 can also promote monoubiquitination of p53; this does not directly cause p53 degradation, but can promote the export of p53 from the nucleus to the cytoplasm especially to the mitochondria and further promote other kinds of modification of p53 [40,41]. *In vitro* studies have shown that DNA damage can destabilize Mdm2 by means of autoubiquitination [39,42] and Mdm2 ubiquitinates MdmX to mark it for proteasomal degradation [43,44]. As discussed above, MdmX does not have appreciable ubiquitin ligase activity, but MdmX has been proposed to inhibit p53 by binding to the N-terminal transcription activation domain of p53 [13]. This binding inhibits p53 activation by hampering the interaction p53 with p300, as acetylation of p53 by p300 can lead to p53 activation and increased transcriptional activity [45]. Consistently, increased endogenous p53 acetylation level is observed in MdmX-null cells. Furthermore, it has been reported that several lysine residues at the C-terminal region of p53 involved in the acetylation are also the same sites of Mdm2-mediated ubiquitination [46]. Thus, it is conceivable that MdmX might indirectly stimulate the Mdm2-mediated ubiquitination of p53 through decreasing the acetylation. Together with the observation that the Mdm2-MdmX heterodimer is a more effective E3 ligase for p53 ubiquitination than Mdm2 alone [26], these data support a model in which the Mdm2-MdmX complex is more efficient in targeting p53 for ubiquitination and degradation.

Ubiquitin-conjugating enzymes (E2s) have a dominant role in determining which of the lysine residues are used for polyubiquitination. Like many other RING domain proteins, the Mdm2 RING domain can promote the transfer of ubiquitin molecules from an E2 conjugating enzyme directly to the lysine residues of the target substrates [47]. Because the E2 enzyme decides the type and length of ubiquitin linkage [48], it is important to identify which E2s are recruited to the Mdm2-MdmX complexes. *In vitro* studies have shown that UbcH5 functions as an E2 enzyme for Mdm2-induced p53 ubiquitination and degradation [49]. Whether UbcH5 functions *in vivo* as the main E2 for Mdm2, or whether there are other E2 enzymes that interact with Mdm2 remains to be determined. A recent study [50] has shown that E2 enzyme in the absence of the appropriate E3 ubiquitin ligase is sufficient to promote the ubiquitination of the substrate. Based on the results of this study, the authors conclude that the main function of E3 ligases include: to specify the lysines to be ubiquitinated, to specify the conformation of ubiquitination, to specify mono versus polyubiquitination, and to define the target region on the substrate to be ubiquitinated.

## PHOSPHORYLATION OF MDM2 AND MDMX

In addition to ubiquitination as a mechanism of controlling Mdm2 and MdmX, the activity of these proteins depends on their phosphorylation status. A number of kinases have been reported to phosphorylate Mdm2 and MdmX at different residues. DNA damage stimulates activation of multiple kinases including ataxia telangiectasia mutated (ATM) [51], checkpoint kinases 1 and 2 (Chk1 and Chk2) [52], DNA-dependent protein kinase (DNA-PK), and c-Abl kinase, which leads to the phosphorylation of both Mdm2 and MdmX [53,54]. Evidence supports the idea that the phosphorylation of Mdm2 by ATM inhibits Mdm2 RING domain homodimerization, which prevents the polyubiquitination of p53 [55]. In addition, ATM and Chk2 have been shown to phosphorylate and destabilize MdmX [56-59]. Another protein that has been shown to regulate Mdm2 and MdmX phosphorylation status is Wip1, which is a phosphatase that can specifically dephosphorylate Mdm2 at Ser395 and MdmX at Ser403 increasing their stability and inhibiting p53 activity [60,61].

The dimerization of Mdm2 and MdmX and Mdm2's E3 ligase function also appear to be regulated by phosphorylation. In an MdmX3SA (Ser-341, -367 and -402 to alanine) knock-in mouse model, Mdm2 retains the ability to bind to MdmX, but is significantly reduced in its capacity to degrade MdmX, resulting in an increase in the concentration of Mdm2-MdmX heterodimers [62]. Thus, the observed defect in p53 stabilization in MdmX3SA mice could be due to the presence of high levels of Mdm2-MdmX complexes. This study also found an approximately 50% reduction in the basal p53 activity in MdmX3SA mice indicating that the stoichiometric balance between Mdm2 and MdmX is crucial for p53 activation and its response to DNA damage stress *in vivo*.

## INHIBITORS TARGETING MDM2 AND MDMX

Although approximately 50% of cancers harbor p53 mutations, the other 50% of cancers retain WT p53, yet they remain uninhibited by the tumor suppression activity of p53. This is generally accomplished through the overexpression of Mdm2 or MdmX by gene amplification or mutation. It has been accepted, at least theoretically, that reactivation or restoration of the p53 function in tumors is a promising cancer therapeutic strategy. Some proposed strategies include repressing the expression of Mdm2, blocking the p53-Mdm2 interaction, and inhibiting the ubiquitin ligase activity of Mdm2 [63,64]. For example, Nutlin, a small molecule that inhibits Mdm2, can trigger cell-cycle arrest and apoptosis and exhibits antitumor efficacy in a murine xenograft model [65]. Several studies also revealed that rational combination of Nutlin-3a and other drugs could potentiate chemotherapy with mitotic inhibitors against cancer and protect normal cells from cytostatic agent [66,67]. However, still several issues have been raised from studies of Nutlin. One of them is the high toxicity of inhibiting Mdm2 by Nutlin. Studies in mice indicate that Mdm2 loss leads to induction of p53 activation and p53-dependent pathologies in both proliferating and quiescent cells, such as erythroid progenitor cells, neurons and smooth muscle cells [68]. Another limitation with Nutlin is that although Nutlin kills cancer cells that express elevated Mdm2, tumor cells overexpressing MdmX have poor response to Nutlin due to its low binding affinity for MdmX compared to Mdm2. As an alternative route for p53 inhibition, overexpression of MdmX in tumor cells has been observed. This will decrease the efficacy for anti-Mdm2-based cancer therapy. Therefore, the development of compounds targeting both Mdm2 and MdmX in tumors retaining WT p53 has become a promising therapeutic goal.

Recently, Bernal *et al.* [69] showed in both *in vitro* and *in vivo* experiments that a “stabilized alpha-helix” of p53 peptide, SAH-p53-8, preferentially inhibits the binding of p53 with MdmX and reduces cancer cell viability, thereby overcomes MdmX-mediated cancer resistance. SAH-p53-8 is derived from the so-called “stapled” peptides SAH-p53 that was designed based on the peptide sequence of the p53 transactivation domain. This peptide show protease resistant combined with increased cellular uptake properties due to a chemical designed strategy termed “hydrocarbon stapling”, which can mimic the biological function of the nature α-helical structure. Co-immunoprecipitation experiments indicate that this peptide can bind to both Mdm2 and MdmX within the cells. Although SAH-p53-8 exhibits a 25-fold greater binding preference for MdmX over Mdm2, it has been shown to have the ability to kill cancer cells that overexpress Mdm2, MdmX, or both of the proteins. More importantly, SAH-p53-8 has been shown to efficiently induce a tumor-suppressive response *in vivo*. The study provides a clue to reactivate p53 tumor suppressor function by synergistically applying Mdm2 and MdmX inhibitors in cancer cells, and affords new therapeutic opportunities for simultaneously inhibiting both Mdm2 and MdmX to restore p53 using drug combinations or dual-inhibitory drugs [70-72]. Thus, efficient rescue of p53 by pharmacological drugs targets Mdm2-MdmX hetero-oligomers is conceptually viable.

## CONCLUDING REMARKS

Over the past decade, considerable progress has been made towards understanding the regulation of p53 by Mdm2 and MdmX, and much of which has come from data obtained from various mouse models. It is generally accepted that the ubiquitination of p53 is a fundamental mechanism of p53 control and that Mdm2 is the principal p53 ubiquitin ligase [36,73]. A study of an Mdm2 RING finger mutant knockin mouse model [12] has shown that Mdm2 is in fact not regulated by autoubiquitination *in vivo*, nor is it capable of blocking p53 activity by binding alone. This is consistent with an earlier report that small molecules that inhibit the E3 ubiquitin ligase activity of Mdm2 can activate p53 [10,74]. However, the exact mechanism underlying the degradation of p53, the regulation of the RING domain of Mdm2, and the role of MdmX in this process is still unclear. Therefore, it is essential to fully understand how the RING domain of Mdm2 regulates p53; whether it is an independent mechanism whereby Mdm2 modifies p53 directly by ubiquitination and degradation, or whether Mdm2 requires MdmX binding in order to regulate p53 activity by the Mdm2-MdmX heterodimer. Recent studies using MdmX RING mutant knockin mouse models can account for part of the story. They show that Mdm2 with an intact RING domain and intrinsic E3 ligase activity are not sufficient for the inhibition of p53 activity in the absence of interaction with MdmX. These studies provide the first *in vivo* evidence that the association of Mdm2 with MdmX, but not the Mdm2 E3 ligase activity, is necessary for p53 control, at least in the developmental stage of mice, which is consistent with previous data based on *in vitro* experiments. Nevertheless, several questions still remain: Whether degradation must occur in order for p53 to be rendered inactive, or whether ubiquitination without degradation is sufficient for the inhibition of p53, how the Mdm2-MdmX heterodimer enables Mdm2 to be more efficiently ubiquitinating p53, and whether the Mdm2-MdmX heterodimer affects p53 ubiquitination. Although much has already been learned about the regulation of p53 by Mdm2 and MdmX, much still remains unknown. Crystal structure studies are needed to further understand at the molecular level how exactly the Mdm2-MdmX-p53 ternary complex is formed and why the Mdm2-MdmX complex is a more efficient E3 ligase complex than Mdm2 alone. The histone acetyltransferase PCAF [75] has been identified as an E3 ubiquitin ligase that mediates the degradation of Mdm2. However, can Mdm2 be ubiquitinated and degraded by an as yet undefined E3 ubiquitin ligase? Under which circumstances is p53 monoubiquitinated and polyubiquitinated? Do Mdm2 and MdmX have additional functions independent of regulating p53? The answers to these questions will be important for understanding the importance of the Mdm2-MdmX heterodimer in tumorigenesis and for determining the feasibility of the Mdm2-MdmX heterodimer as a target for cancer therapy.
